# Avicin D, a Plant Triterpenoid, Induces Cell Apoptosis by Recruitment of Fas and Downstream Signaling Molecules into Lipid Rafts

**DOI:** 10.1371/journal.pone.0008532

**Published:** 2009-12-31

**Authors:** Zhi-Xiang Xu, Tian Ding, Valsala Haridas, Fiona Connolly, Jordan U. Gutterman

**Affiliations:** 1 Department of Systems Biology, The University of Texas M. D. Anderson Cancer Center, Houston, Texas, United States of America; 2 Division of Hematology and Oncology, The University of Alabama at Birmingham, Birmingham, Alabama, United States of America; 3 Department of Molecular Pathology, The University of Texas M. D. Anderson Cancer Center, Houston, Texas, United States of America; 4 Genscript Inc., Piscataway, New Jersey, United States of America; INSERM U567, Institut Cochin, France

## Abstract

Avicins, a family of triterpene electrophiles originally identified as potent inhibitors of tumor cell growth, have been shown to be pleiotropic compounds that also possess antioxidant, anti-mutagenic, and anti-inflammatory activities. We previously showed that Jurkat cells, which express a high level of Fas, are very sensitive to treatment with avicins. Thus, we hypothesized that avicins may induce cell apoptosis by activation of the Fas pathway. By using a series of cell lines deficient in cell death receptors, we demonstrated that upon avicin D treatment, Fas translocates to the cholesterol- and sphingolipid-enriched membrane microdomains known as lipid rafts. In the lipid rafts, Fas interacts with Fas-associated death domain (FADD) and Caspase-8 to form death-inducing signaling complex (DISC) and thus mediates cell apoptosis. Interfering with lipid raft organization by using a cholesterol-depleting compound, methyl-β-cyclodextrin, not only prevents the clustering of Fas and its DISC complex but also reduces the sensitivity of the cells to avicin D. Avicin D activates Fas pathways independent of the association between extracellular Fas ligands and Fas receptors. A deficiency in Fas and its downstream signaling molecules leads to the resistance of the cells to avicin D treatment. Taken together, our results demonstrate that avicin D triggers the redistribution of Fas in the membrane lipid rafts, where Fas activates receptor-mediated cell death.

## Introduction

The Fas death receptor (CD95 or APO-1) conveys apoptotic signals through binding to its cognate ligand, FasL (CD95L), making the interaction between FasL and Fas a potential tumor-fighting target. Unfortunately, the putative clinical antitumor action of FasL cannot be reached because of severe liver toxicity due to the high expression of Fas on the surface of hepatocyte [1]. Recent evidence for a FasL-independent activation of Fas suggests that the death receptor can also be activated intracellularly, in the absence of its ligand [2]. Unraveling the mechanisms could provide the basis for novel therapeutic strategies for various diseases and could aid in the development of new compounds able to exploit cytoplasmic triggers of apoptosis for clinical use. This is of importance in apoptosis-deficient disorders such as cancer [2] and autoimmune diseases [3].

Fas-mediated apoptosis involves translocation of Fas–and its downstream signaling molecules into lipid rafts, a process that can be pharmacologically modulated [2]. FasL-independent clustering of Fas in membrane rafts generates high local concentrations of death receptors, providing scaffolds for coupling the adaptor and effector proteins involved in Fas-mediated apoptosis [4]. Thus, lipid rafts act as the linchpin from which a potent death signal is launched, suggesting a promising new target for anticancer therapeutics. These findings have revealed a novel framework for the development of more targeted therapies leading to intracellular Fas activation and for the recruitment of downstream signaling molecules into Fas-enriched lipid rafts [2], [3], [4].

Avicins, a family of triterpene electrophiles, selectively inhibit the growth of various tumor cells [5], [6], [7], [8]. Avicins activate the intrinsic caspase pathway to induce apoptosis by direct perturbation of mitochondria, and downregulate a group of pro-survival, anti-apoptotic proteins, including HSP-70, XIAP, PI3K, AKT, and NF-κB [6], [7], [8], [9], [10]. We further showed in a recent report that avicins can induce not only apoptotic cell death but also autophagic programmed cell death by depletion of the cell energy supply, suggesting a potential therapeutic application of avicins in apoptosis-resistant cancers [11]. In addition to their cytotoxic properties, avicins possess cyto-protective effects against non-transformed cells, in part related to the activation of the transcription factor Nrf-2 [12]. Activation of proteins downstream of Nrf2, such as glutathione peroxidase, heme oxygenase, and thioredoxin reductase, accounts in part for the antioxidant, anti-mutagenic and tissue protective effects of avicins in vitro and in vivo [9], [12], [13], [14]. The studies indicated that avicins, multifunctional compounds, not only inhibit tumor cell growth but may play a role in the maintenance of cellular homeostasis as well.

Our previous findings showed that Jurkat cells expressing high levels of Fas are very sensitive to avicin treatment [6]. Thus, we hypothesized that avicins may induce apoptosis by activation of the Fas pathway. In the present study, with a series of cell lines deficient in cell death receptors, we demonstrated that upon avicin D treatment, Fas translocates to lipid rafts, where it associates with Fas-associated death domain (FADD) and Caspase-8 to form death-inducing signaling complex (DISC), and thus mediates cell apoptosis.

## Materials and Methods

### Cell Lines

U2OS, Daudi, NB4, and parental Jurkat Human cell lines were purchased from ATCC. U2OS (osteosarcoma) cells were grown in Dulbecco modified Eagle medium (DMEM) containing 10% fetal bovine serum (FBS) in a humidified incubator containing 5% CO2 at 37°C. The human leukemia cell lines, Daudi, NB4, and parental Jurkat cells and those deficient in Fas [2], FADD [15], Caspase-8 [15], and receptor interacting protein (RIP) [16] were cultured in RPMI 1640 medium containing 10% FBS.

### Antibodies and Chemicals

Antibodies against Caspase-3, Caspase-7, FADD, RIP, Poly (ADP-ribose) polymerase (PARP), and Bid were purchased from BD Biosciences (San Diego, CA). Anti-Fas (human, activating) antibody (clone CH11) and anti-Fas (human, neutralizing) antibody (clone ZB4) were purchased from Millipore (Billerica, MA). The activating anti-Fas antibody was used to induce Jurkat cell apoptosis at 10 µg/ml. The neutralizing anti-Fas antibody (50 ng/ml) was used to block Jurkat cell apoptosis. Caspase-8 and inhibitor of caspase-activated DNase (ICAD)/DNA-fragmentation factor 45 (DFF-45) were purchased from Upstate Biotechnology Inc. (Lake Placid, NY). Anti-tumor necrosis factor α (TNFα) and anti-cellular FLICE-inhibitory protein (c-FLIP) antibodies were purchased from Santa Cruz Biotechnology (Santa Cruz, CA). Anti-α-tubulin monoclonal antibody and Anti-β-actin polyclonal antibody were purchased from Sigma Aldrich (St. Louis, MO) and Bethyl Laboratories (Montgomery, TX), respectively. Methyl-β-cyclodextrin, fluorescein isothiocyanate (FITC)-cholera toxin B subunit (FITC-CTXb), and CTXb subunit conjugated to horseradish peroxidase were purchased from Sigma Aldrich. Avicin D and avicin G were purified from A. victoriae root extracts as described in our recent report [17].

### Cell Death and Viability Assay

Cell death was examined by the cell death enzyme-linked immunosorbent assay (ELISA) according to the manufacturer's protocol of Roche Diagnostics (Pleasanton, CA) or by trypan blue exclusion kit from Sigma-Aldrich (St. Louis, MO) [18]. Cell viability was measured by a CellTiter-Glo Luminescent Cell Viability Assay kit from Promega Corp (Madison, WI) per the manufacturer's protocol and by trypan blue exclusion assay [11].

### Flow Cytometry Analysis of DNA Content

For flow cytometry (FCM) analysis of the DNA content, cells were treated, collected, and fixed in 70% ethanol for at least 24 h. The cells were then resuspended in phosphate-buffered saline (PBS), treated with 2 N HCl containing 0.2 mg/ml pepsin, and neutralized with 0.1 M sodium tetraborate. Following washes with PBS, cells were incubated in RNase A (0.5 mg/ml) and propidium iodide (50 µg/ml) diluted to an equal concentration and analyzed by FCM [18].

### Isolation of Lipid Rafts by Discontinuous Sucrose Gradient Centrifugation

Lipid rafts were isolated by using lysis conditions and centrifugation on discontinuous sucrose gradients as previously reported [2]. Briefly, 1×108 Jurkat cells were washed with ice-cold PBS and lysed for 30 min on ice in 1% Triton X-100 in TNEV buffer (10 mM Tris-HCl, pH 7.5, 150 mM NaCl, 5 mM EDTA, 1 mM sodium orthovanadate) containing 1 mM phenylmethylsulfonyl fluoride. Cells were then homogenized with 10 strokes in a Potter-Elvehjem tissue grinder (Vernon Hills, Illinois). Nuclei and cellular debris were pelleted by centrifugation at 1000 rpm for 8 min. Then, 1 mL of cleared supernatant was mixed with 1 mL of 85% sucrose in TNEV and transferred to the bottom of a Beckman 14×95-mm centrifuge tube. The diluted lysate was overlaid with 6 mL of 35% sucrose in TNEV and, finally, with 4 mL of 5% sucrose in TNEV. The samples were centrifuged in an SW40 rotor at 38 000 rpm for 18 h at 4°C in a Beckman Optima LE-80K ultracentrifuge (Beckman Instruments, Palo Alto, CA), and then 0.75-mL fractions were collected from the top of the gradient.

To determine the location of lipid rafts and Fas and its downstream targets in the discontinuous sucrose gradient, 25 µL of the individual fractions were subjected to 12% sodium dodecyl sulfate-polyacrylamide gel electrophoresis (SDS-PAGE) and immunoblotted with different antibodies. The location of the lipid rafts was determined by a dot blot using the CTXb subunit conjugated to horseradish peroxidase.

### Immunofluorescent Staining and Fluorescent Microscope

Immunofluorescent staining was performed in accordance with procedures described previously [19]. Briefly, Jurkat cells were cyto-spun to a microscope slide with the Shandon CytoSpin III Cytocentrifuge (Ramsey, Minnesota). The cells were then fixed in 4% neutral paraformaldehyde for 30 min, washed in PBS, permeabilized in a solution containing 0.1% Triton X-100/0.05% NP40/PBS, and blocked with 1% bovine serum albumin (BSA). Cells were incubated with anti-Fas antibody and CTx B-FITC for 2 h and then allowed to interact with a rhodamine conjugated secondary antibody for 1 h at room temperature, followed by staining of the DNA with 4′,6-diamidino-2-phenylindole (DAPI) for 5–10 min. Fluorescent signaling was observed with a Nikon Eclipse TE2000-E fluorescent microscope.

### Immunoblotting

Cell lysis and immunoblotting were performed as described previously [19]. A total of 50 µg of protein was used for the immunoblotting (unless otherwise indicated). β-actin or α-tubulin was used for the loading control.

## Results

### Avicin D Activates Cell Apoptosis Mediated by Fas–Caspase-8

**Figure 1:**
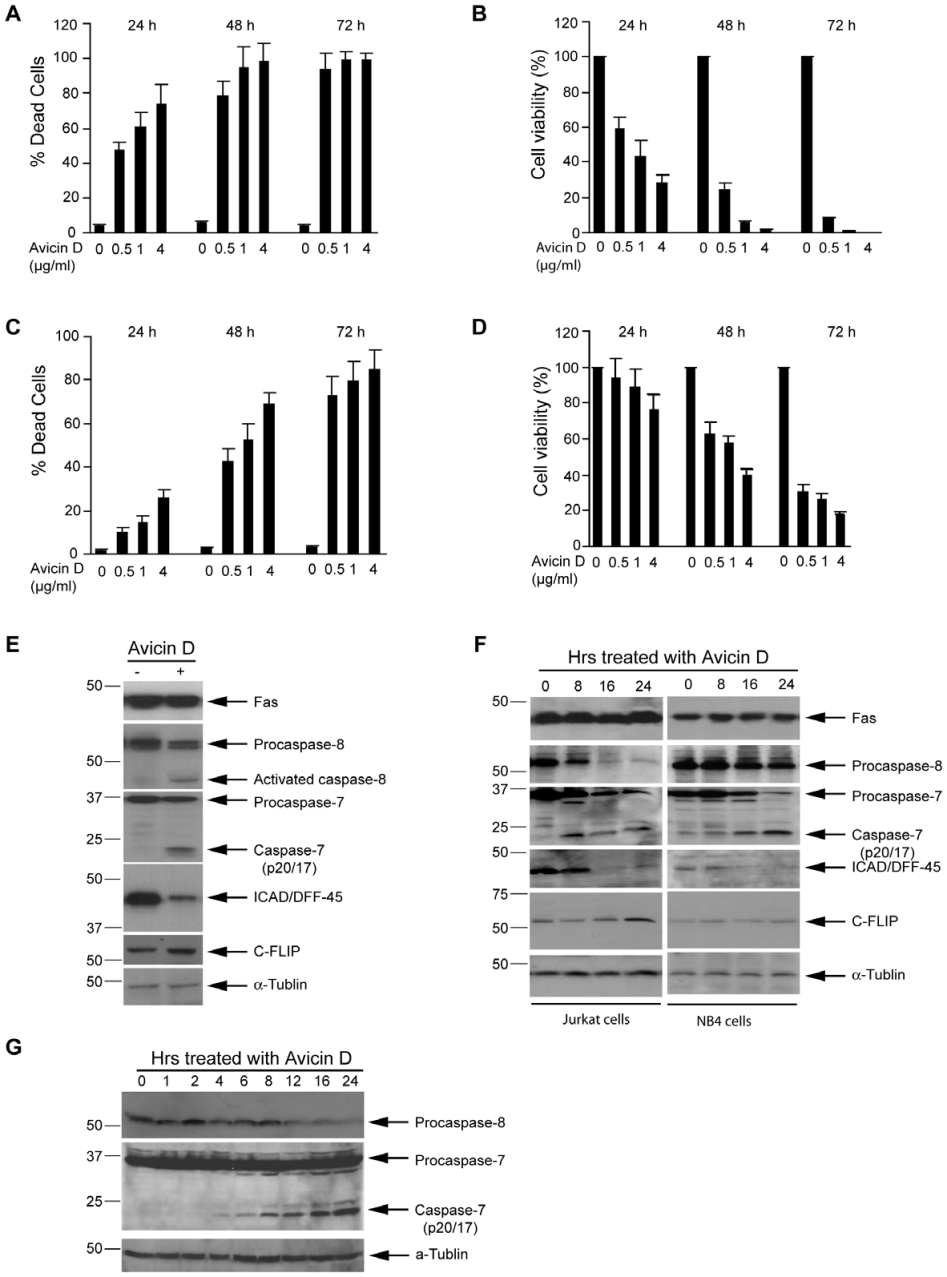
Avicin D activates Caspase-8–mediated cell death. Proliferating Jurkat cells were exposed to different doses of avicin D for 24–72 h. A. Cell death was measured by trypan blue exclusion assay. The number of dead cells was counted as a percentage of total cells. B. Cell viability was measured by a CellTiter-Glo Luminescent Cell Viability Assay kit. Data are shown as mean±S.D. (n = 5). The number of surviving cells was counted as a percentage of total cells. C and D. Cell death and cell viability were measured in NB4 cells exposed to 0–4 µg/ml of avicin D for 24–48 h as described in the Methods. Data are shown as mean±S.D. (n = 3). The number of dead or surviving cells was counted as a percentage of total cells. E. Proliferating Jurkat cells were exposed to 2 µg/ml of avicin D for 24 h. Cleavage of Caspase-8 induced by avicin D was analyzed by Western blot. 50 µg of total protein were loaded in each lane. α-Tubulin was detected to serve as an internal control. F. Time-dependent activation of Caspase-8 and apoptotic regulators in avicin D-treated Jurkat and NB4 cells. G. Time-dependent activation of Caspase-8 and apoptotic regulators in avicin D-treated U2OS cells.

To investigate whether avicins can induce cell death via activation of the death receptor–Caspase-8 pathway, we exposed Jurkat cells and a promyelocyte leukemia cell line, NB4 cells, to various concentrations of avicin D for 24–72 h and analyzed the viability and activation of the Caspase-8 pathway. Consistent with our previous reports [6], avicin D treatment resulted in substantial cell death (Figure 1A, C). After exposure to 0.5 µg/ml of avicin D for 72 h, 70% of the NB4 cells and 90% of the Jurkat cells committed to cell death (Figure 1A, C), and cell viability was significantly inhibited (Figure 1B, D). Avicin D induced cell death and decreased cell viability in a time- and dose-dependent manner (Figure 1A–D). Interestingly, avicin D treatment also led to an elevated cleavage of Caspase-8, an important indicator of death receptor-induced apoptosis (Figure 1E). The activation of Caspase-7 and ICAD/DFF-45, two downstream targets of the death receptor–Caspase-8 pathway [20], was also induced by this treatment with avicin D (Figure 1E). The activation of Caspase-8 and its downstream targets was induced as early as 8 h after avicin D treatment (Figure 1F). The time-dependent activation of Caspase-8 by treatment with avicin D was also confirmed in a human osteosarcoma cell line, U2OS (Figure 1G). Taken together, these data suggest that avicin D-mediated cell death is associated with the activation of the death receptor–Caspase-8 pathway.

### Cell Apoptosis Induced by Avicin D and Mediated by Fas–Caspase-8 Is Independent of Extracellular Fas Activation

**Figure 2:**
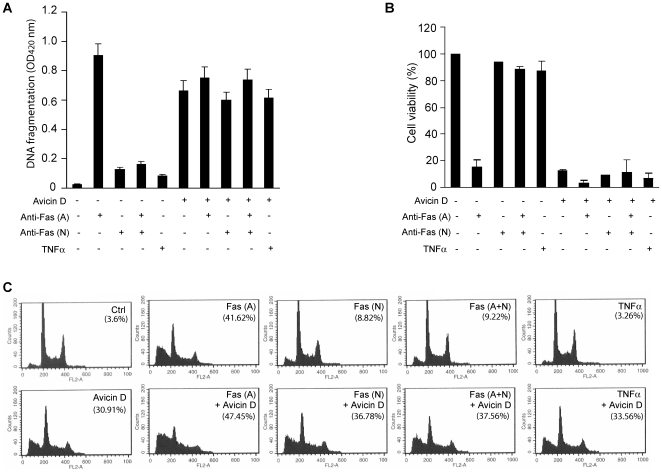
Avicin D-induced cell death is not via an extracellular mechanism. Proliferating Jurkat cells were pretreated with activating anti-Fas antibody (A), neutralizing anti-Fas antibody (N), activating and neutralizing anti-Fas antibody (A+N), or TNFα for 2 h, followed by avicin D (2 µg/ml) for 24 h. A. Cell death was quantified using a cell death ELISA showing the enrichment of nucleosomes in the cytoplasmic fraction of Jurkat cells. Values represent the mean±S.D. (n = 3). B. Cell viability was measured by a CellTiter-Glo Luminescent Cell Viability Assay kit. Data are shown as mean±S.D. (n = 4). The number of surviving cells was counted as a percentage of total cells. C. Apoptosis and cell-cycle distribution of Jurkat cells treated with avicin D and/or anti-Fas antibodies and TNFα. Flow cytometry (FCM) analysis was carried out to detect sub-G1 (apoptotic cells) and other cell-cycle distributions. The numbers in parenthesis are the percentage of sub-G1. Representative FCM graphs of three experiments are shown.

To determine whether avicin D-induced Caspase-8 activation and cell apoptosis are associated with extracellular Fas activation, we pretreated Jurkat cells with neutralizing anti-Fas antibody for 2 h. Neutralizing anti-Fas antibody blocks extracellular activation signaling, such as that of Fas ligands and activating antibodies, and inhibits the apoptosis induced by these factors. We then treated the cells with avicin D. The enrichment of nucleosomes from the cytoplasmic fraction (DNA fragmentation) in Jurkat cells treated with the neutralizing anti-Fas antibody and avicin D existed no significant difference compared to that of avicin D treatment alone, indicating that the neutralizing anti-Fas antibody did not block avicin D-induced cell death (Figure 2A). In contrast, the neutralizing anti-Fas antibody completely blocked the activation of cell death as mediated by cytotoxic anti-Fas CH11 antibodies (Figure 2A). Consistent with the results of the DNA fragmentation assay, neutralizing anti-Fas antibody could not improve the viability of cells treated with avicin D either (Figure 2B).

Flow cytometry analysis of sub-G1/G0 cells is an important indicator by which to divide apoptotic cells from cells undergoing other types of death. To confirm the apoptotic nature of the cell death induced by avicin D and neutralizing anti-Fas antibodies is not sufficient to block avicin D's effects, we pre-treated Jurkat cells with 50 ng/ml neutralizing anti-Fas antibody for 2 h. We then treated the cells with either avicin D or activating anti-Fas antibody. Although the neutralizing anti-Fas antibody did in fact substantially reduce the cell apoptosis (distribution of sub-G1/G0 phase cells) induced by activating anti-Fas antibody, it did not block the avicin D-induced cell apoptosis (Figure 2C). Together, our results suggest that avicin D-induced cell apoptosis mediated by Fas–Caspase-8 is independent of extracellular Fas activation.

### Avicin D Induces the Clustering of Fas into Lipid Rafts

**Figure 3:**
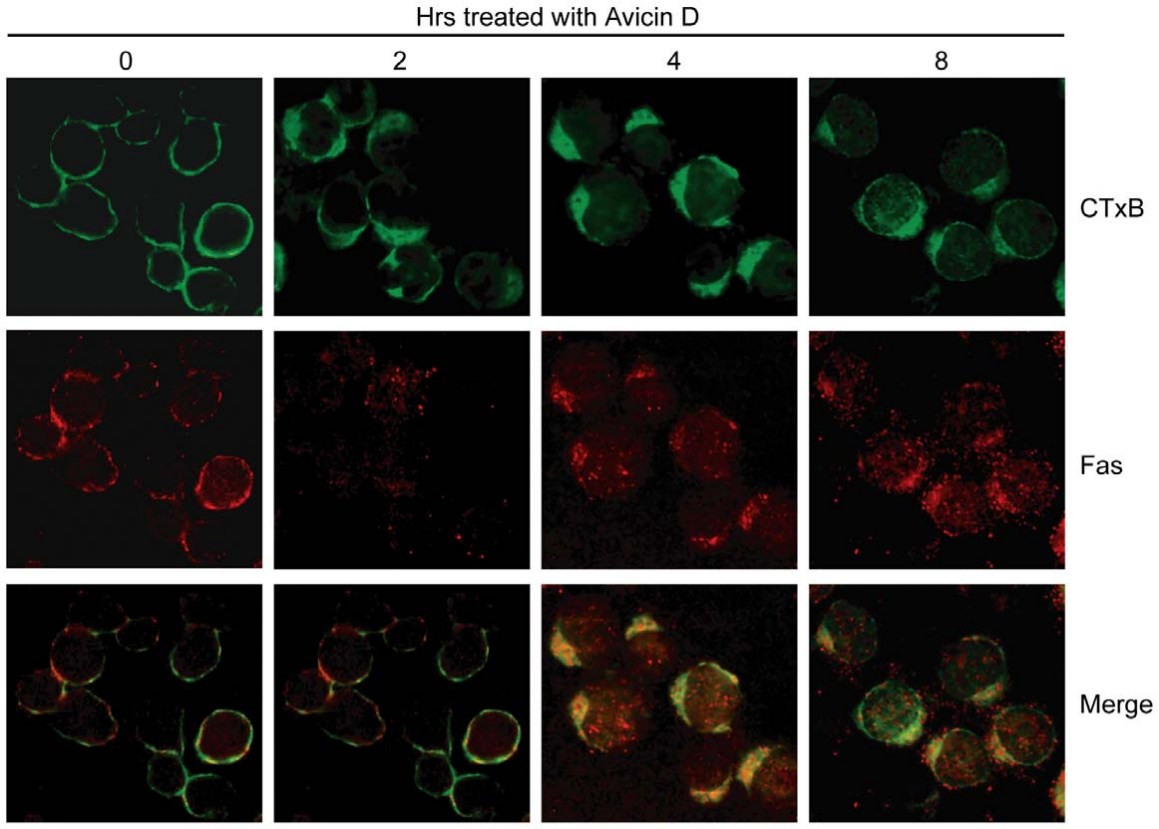
Avicin D induces clustering of Fas in lipid rafts. Jurkat cells were treated with 2 µg/ml of avicin D for 0–8 h. After the treatment, the cells were fixed and stained with FITC-CTxB subunits to identify lipid rafts (green fluorescence) and with anti-Fas antibodies to identify Fas (red fluorescence). Area of colocalization between membrane rafts and Fas in the merge panels is yellow.

Lipid rafts on the cell membrane have been reported to play an important role in the activation of death receptors [3], [21], [22]. To determine whether lipid rafts might serve as a platform for avicin D to induce Fas–Caspase-8 activation and apoptosis, we exposed Jurkat cells to 2 µg/ml of avicin D for 0–8 h and detected the distribution of lipid rafts and translocation of Fas by immunofluorescence. Membrane rafts were assessed by the FITC-CTx B subunit marker that binds ganglioside GMi, which mainly exists in rafts [23]. In the control experiment of Jurkat cells without any treatment, lipid rafts distributed on the cell membrane evenly (Figure 3A). Fas receptors located on the cell membrane showed less colocalization with lipid rafts (Figure 3A′, A″). In contrast, following treatment with avicin D, lipid rafts clustered and formed a “cap-like” structure at one pole of the cells, which happened within 2 h after the treatment (Figure 3B–D). Avicin D treatment also led to the aggregation of Fas although it occurred after the clustering of lipid rafts (Figure 3B′–D′). Importantly, engaged Fas co-localized with lipid rafts (Figure 3B″–D″). In addition to Fas, Jurkat cells also express several other types of death receptors, such as TNFR1 and TRAIL/DR5. We next questioned whether avicin D could also trigger other death receptors translocated to lipid rafts. Our results showed that avicin D did not induce the engagement and translocation of TNFR1 and TRAIL/DR5 to lipid rafts (data not shown), which is consistent with the component analysis of lipid rafts extracted with a discontinuous sucrose density gradient centrifugation (see below for details). Collectively, our results suggest that avicin D induces the clustering of lipid rafts and specifically mediates the recruitment of Fas into lipid rafts.

### Avicin D-Induced Apoptosis Is Dependent upon Lipid Rafts

**Figure 4:**
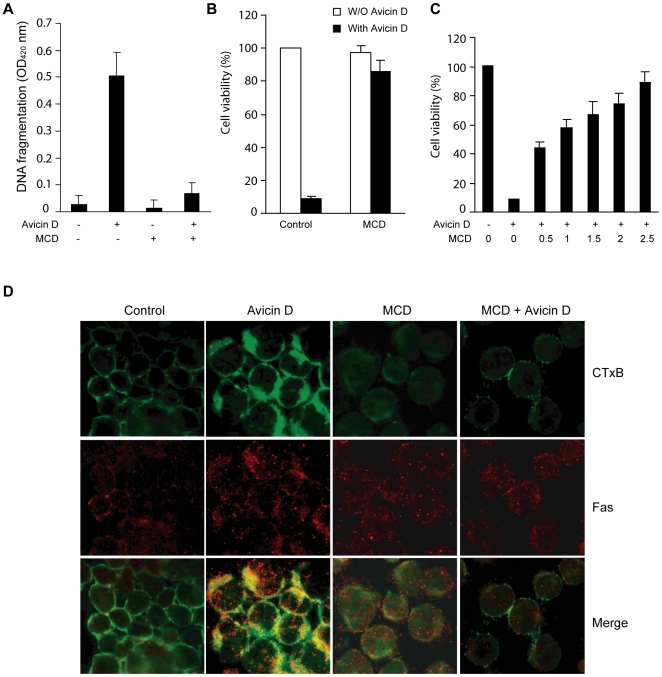
Avicin D-induced apoptosis is lipid raft-dependent. (A & B) Proliferating Jurkat cells were pretreated with 2.5 mg/ml of MCD for 1 h, followed by 2 µg/ml of avicin D for 24 h. A. Cell death was quantified using a cell death ELISA showing enrichment of nucleosomes in the cytoplasmic fraction of Jurkat cells. Values represent the mean±S.D. (n = 3). B. Cell viability was determined by trypan blue exclusion assay. The number of surviving cells was counted as a percentage of total cells. C. MCD inhibits the activity of avicin D in a dose-dependent manner. Increasing concentrations of MCD were applied to Jurkat cells. One hour after treatment with MCD, 2 µg/ml of avicin D were added to the medium. Cell viability was determined by trypan blue exclusion assay after 24 h. Jurkat cells without treatment with MCD and avicin D were set as 100%. D. Jurkat cells were pretreated with 2.5 mg/ml MCD for 1h, followed by 2 µg/ml avicin D for 8 h. The cells were then fixed and stained with FITC-CTxB subunits to identify rafts (green fluorescence) and with anti-Fas antibody to identify Fas (red fluorescence). Area of colocalization between membrane rafts and Fas in the merge panels is yellow.

To further confirm that the apoptosis induced by avicin D in Jurkat cells is associated with the engagement and recruitment of Fas into lipid rafts, we investigated cell apoptosis and lipid raft formation in Jurkat cells pretreated with an agent that disrupts lipid rafts, methyl-β-cyclodextrin (MCD) [24]. The disruption of lipid rafts caused by a 1 h pretreatment with 2.5 mg/ml MCD prevented avicin D-induced apoptosis (Figure 4A). Pretreatment with MCD also remarkably increased the viability of avicin D-treated Jurkat cells (Figure 4B). MCD blocked avicin D-induced cell apoptosis and extended cell viability in a dose-dependent manner, with the maximum effect being seen after treatment with 2.5 mg/ml of MCD (approximately 80% reversal of apoptosis) (Figure 4C and other data not shown). MCD disorganized the clustering of lipid rafts that had been induced by avicin D and disrupted the aggregation and translocation of Fas into lipid rafts (Figure 4D). To eliminate the possibility that extracellular interactions between MCD and avicin D were reducing the cytotoxicity of avicin D, we pre-exposed Jurkat cells and Daudi cells to 2.5 mg/ml MCD for 8 h, and avicin D was supplied after the MCD was washed off the cells. We found that pre-treatment with MCD was sufficient to block avicin D-induced cell apoptosis. Cell apoptosis (sub-G1) was significantly reduced in both Jurkat and Daudi cells (data not shown). Collectively, our results suggest that the clustering of Fas-containing lipid rafts is required for avicin D to induce apoptosis.

### Cell Membrane Integrity Is Necessary for Avicin D to Function

**Figure 5:**
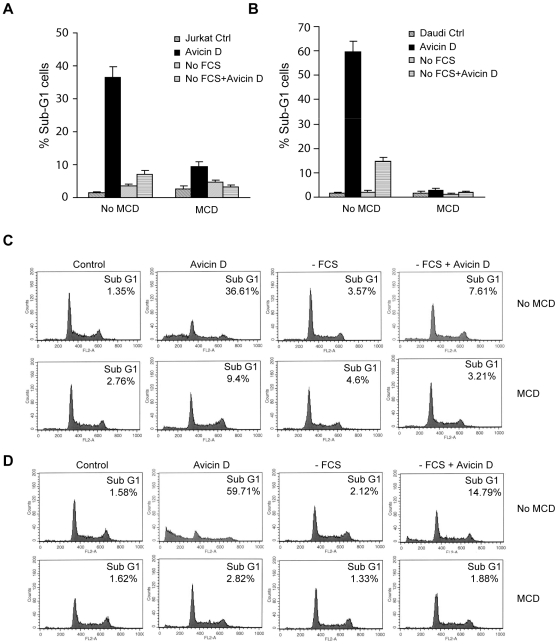
The integrity of the cell membrane is needed for the function of avicin D. Proliferating Jurkat (A, C) and Daudi (B, D) cells were pretreated with methyl-β-cyclodextrin (MCD) for 8 h. After washing away the MCD, cells were exposed to 2 µg/ml Avicin D for 24 h in DMEM medium with or without 10% FCS. Cells were fixed and stained with PI. Flow cytometry analysis was carried out to detect sub-G1 (apoptotic cells) and cell cycle distribution. A. Percentage of sub-G1 Jurkat cells after the treatment. Data shown are means±S.D. of three independent experiments. B. Percentage of sub-G1 Daudi cells after the treatment. Data shown are means±S.D. of three independent experiments. C. Representative FCM graphs of Jurkat cells after the treatment. D. Representative FCM graphs of Daudi cells after the treatment.

To further confirm that the integrity of cell membrane, where lipid rafts are located, is required for avicin D-mediated cell apoptosis, we cultured Jurkat and Daudi cells in serum-free medium for 24 h, then treated the cells with avicin D. Serum provides cells with cholesterol to build up lipid rafts in cell membranes. As shown in Figure 5A and 5C, 36% of the Jurkat cells treated with 2.0 mg/ml avicin D for 24 h in regular cell culture medium (10% serum) committed to apoptosis (distributed in the sub-G1 phase). In contrast, only 7% of the cells treated with avicin D in serum-free medium exhibited apoptosis, suggesting that depletion of serum (cholesterol) could block avicin D-mediated cell apoptosis. By using the avicin D-sensitive human lymphoma cell line, Daudi, we obtained the same results as shown in the Jurkat cells (Figure 5B–D). Taken together, our results suggest that the integrity of the cell membrane with functional lipid rafts plays an important role in avicin D-induced cell apoptosis.

### Avicin D Recruits Fas and Its Downstream Signaling Molecules into Lipid Rafts

**Figure 6:**
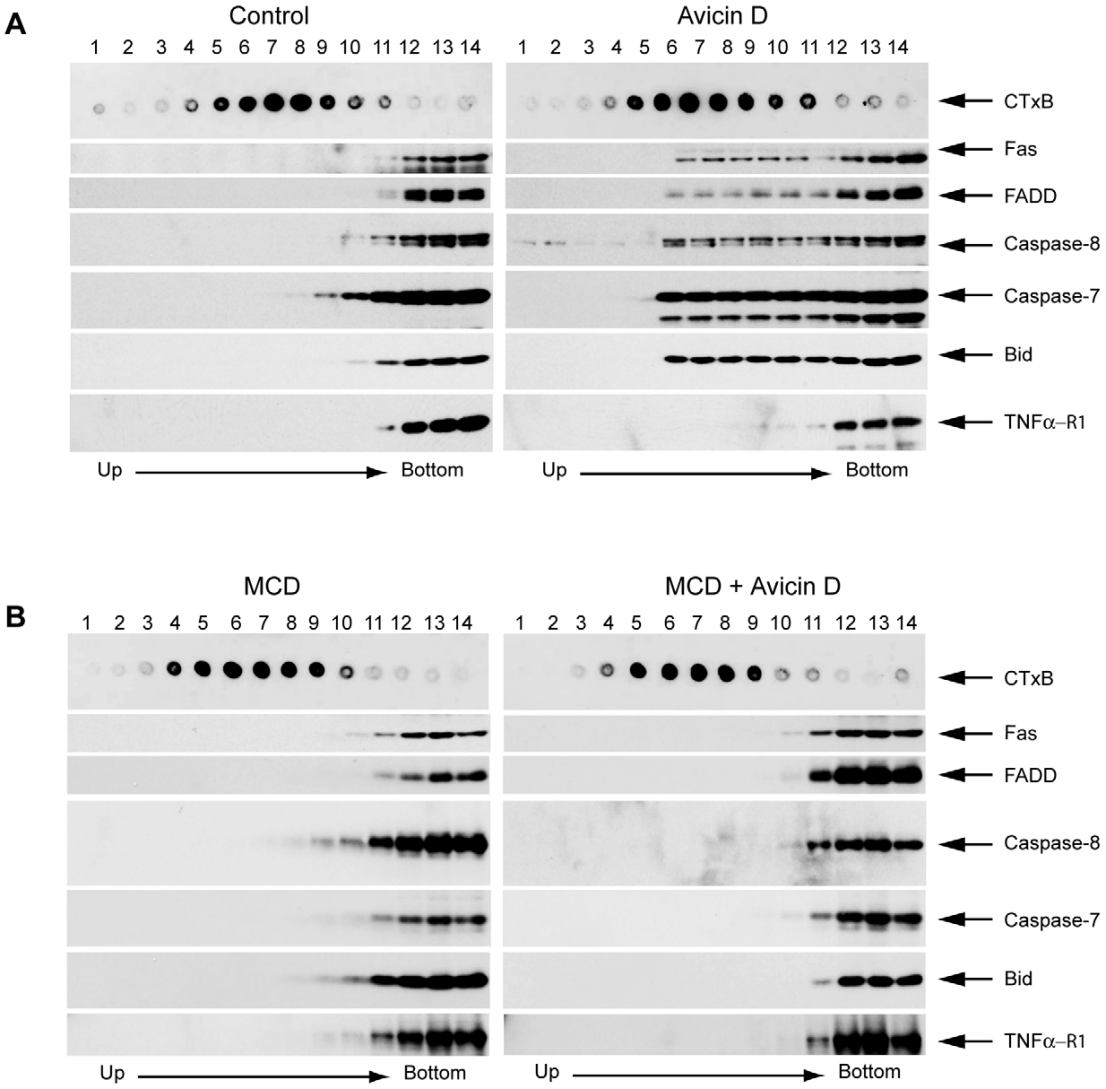
Avicin D recruits Fas and its downstream signaling molecules into lipid rafts. Proliferating Jurkat cells were pretreated with or without 2.5 mg/ml MCD for 1 h, followed by 2 µg/ml Avicin D for 24 h. After the treatment, the cells were lysed in 1% Triton X-100 and subjected to discontinuous sucrose density gradient centrifugation. Components in each fraction were analyzed by Western blot. Location of lipid rafts was determined by a dot blot using CTxB subunits conjugated to horseradish peroxidase. A. Components of lipid rafts in control and avicin D-treated Jurkat cells. B. Components of lipid rafts in MCD with/without avicin D-treated Jurkat cells.

To further confirm the translocation of Fas and its downstream signaling molecules into lipid rafts, we isolated membrane rafts in both untreated and avicin D-treated Jurkat cells in the presence or absence of MCD. Lipid rafts were isolated based on their insolubility in Triton X-100 detergent and buoyant density on sucrose density gradients. The location of the lipid rafts was determined by horseradish peroxidase-conjugated CTx B subunits that bind ganglioside GMi, which mainly exists in rafts [22], [23]. As shown in Figure 6A, GM1 was enriched in fractions 5–9 of the sucrose gradient. In control cells, Fas, FADD, Caspase-8, Caspase-7, Bid, and TNFα-R1 were mainly in the soluble parts of the isolate (fractions 10–14) and were not co-fractioned with GM1, suggesting that these components are not in lipid rafts normally. After treatment with avicin D, however, Fas, FADD, Caspase-8, Caspase-7, and Bid were recruited into the lipid raft region (fractions 5–9) of the sucrose gradient (Figure 6A, right panels). Interestingly, two additional major death receptors, TNFα-R1 and DR5, were not translocated to rafts after the avicin D treatment (Figure 6A and other data not shown), suggesting that the translocation of Fas and its downstream targets were a specific process triggered by avicin D.

In contrast, pretreatment with 2.5 mg/ml of MCD completely abrogated the translocation of the apoptotic signaling molecules regardless of the presence or absence of avicin D (Figure 6B), which further supports our conclusion that avicin D-induced apoptosis is dependent upon lipid rafts and that the integrity of these rafts plays a critical role in avicin D-mediated cell death.

### Aberrations in Fas and Its Downstream Signaling Molecules Abrogate the Effects of Avicin D

**Figure 7:**
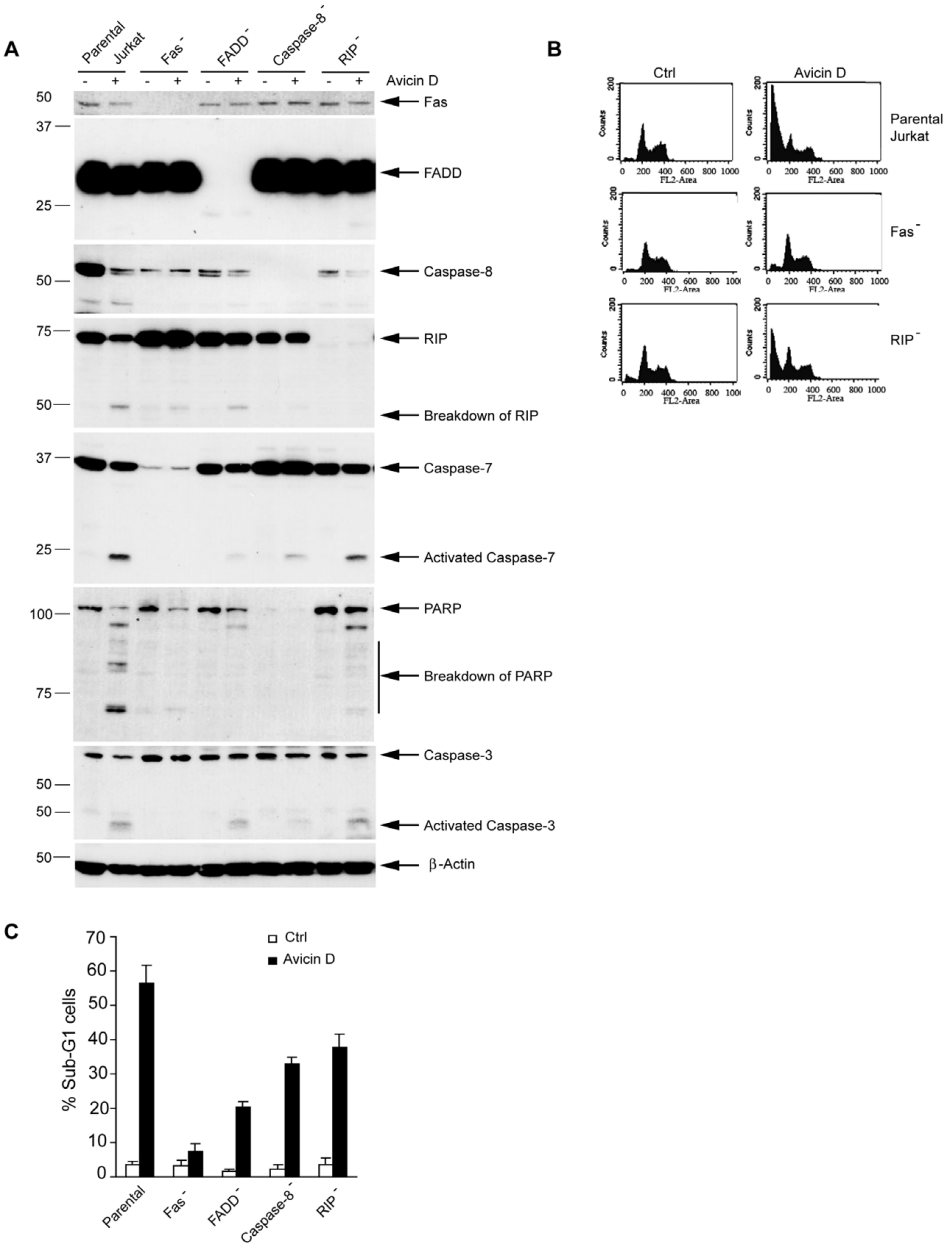
Aberrations in Fas and its downstream signaling molecules abrogate the effects of avicin D. Parental Jurkat cells and Fas-, FADD-, Caspase-8-, and RIP-deficient Jurkat cells were treated with 2 µg/ml of Avicin D for 24 h. A. Detection of apoptotic molecules by Western blot. 50 ìg of total protein were loaded in each lane. β-Actin was detected to serve as an internal control. B. The percentage of apoptotic cells. After the treatment, the cells were fixed and stained with PI. Flow cytometry (FCM) analysis was performed to detect sub-G1 (apoptotic cells). C. Representative FCM graphs of parental, Fas-deficient, and RIP-deficient Jurkat cells.

Since Fas-mediated apoptosis plays an important role in avicin D-induced apoptosis, we next questioned whether depletions of Fas or its downstream targets would reduce the effects of avicin D on cell death. Parental and Fas-, FADD-, Caspase-8–, and RIP-deficient Jurkat cells were exposed to 2 µg/ml of avicin D for 24 h. Treatment with avicin D led to a remarkable activation of Caspase-7, Caspase-3, and PARP in parental Jurkat cells (Figure 7A). By contrast, deficiency of Fas [2], FADD [15], Caspase-8 [15], and RIP [16] reduced the activation of Caspase-7, Caspase-3, and PARP mediated by avicin D (Figure 7A), thus reduced avicin D-mediated apoptosis substantially (Figure 7B-C). Interestingly, in the Fas-FADD-Caspase-8 cell death pathway, the further upstream the deficiency happens, the more resistant the cells are to treatment with avicin D (Figure 7B–C). These results unambiguously prove that Fas and its downstream signaling targets are critical mediators of avicin D-induced cell death.

## Discussion

Lipid rafts are enriched membrane microdomains that contain high concentrations of cholesterol and glycosphingolipids, which are resistant to solubilization by detergents and represent areas of reduced fluidity plane of the lipid bilayer [23], [25]. A variety of proteins, especially those involved in cell signaling, have been shown to translocate into lipid rafts. As a result, lipid rafts are thought to be involved in the regulation of signal transduction.

Experimental evidence suggests that there are probably several different mechanisms through which rafts control cell signaling. For example, rafts may contain incomplete signaling pathways that are activated when a receptor or other required molecule is recruited into the raft. Rafts may also be important in limiting signaling, either by physical sequestration of signaling components to block nonspecific interactions, or by suppressing the intrinsic activity of signaling proteins present within the rafts.

Death receptors, which include Fas (CD95), TNFR1, and TRAIL (R2/DR5) [20], [26], are an important family of proteins that locate on the cell membrane. While the roles of lipid rafts in the function of many membrane receptors have been increasingly clear [4], [24], [27], [28], their importance to death-receptor signaling remains controversial. The discrepancies in the models proposed by different groups focus on whether death receptors are recruited to the lipid rafts and how the death receptors are organized in the lipid rafts if they are indeed aggregated therein.

Fas triggers cell death through the presence of a death domain in its cytoplasmic portion after receptor engagement with FasL or agonistic anti-Fas antibodies [29]. Stimulation of Fas results in the oligomerization of its receptors and the recruitment of the adaptor molecule FADD through the interaction of death domains from both parts, which results in the recruitment of procaspase-8 and the formation of DISC. The proximity of procaspase-8 molecules in the DISC drives its activation by self-cleavage, triggering downstream effector caspases and leading to apoptosis [20], [29]. Thus, formation of membrane platforms where Fas molecules are brought together may increase DISC formation and, therefore, potentiate Fas signaling. In this regard, lipid rafts may provide microdomains for the interaction of Fas cell death complexes and play a role in mediating Fas-induced cell death.

Our results showed that avicin D induce caspase-8-mediated cell apoptosis. Theoretically, activation of Caspase-8 may be mediated through Fas, TNFR1, or TRAIL-R2/DR5 receptors pathways [20]. Avicin D-induced activation of Caspase-8 may involve some or all of these receptors. It is known that stimulation of TNFR1 induces the activation of caspase-8 and NF-κB simultaneously. Our previous study reported that treatments with avicins lead to the inhibition of NF-κB and its associated functions [9]. Thus, avicin D-induced activation of caspas-8 is unlikely through the stimulation of TNFR1. In addition, our functional assay showed that avicin D exerted a synergistic effect with TNFα and TRAIL (data not shown), indicating that the action of avicin D is independent of TNFR1 and TRAIL-R2/DR5. Therefore, we conclude that avicin D-induced Caspase-8 activation and cell apoptosis are mainly associated with Fas receptors.

Importantly, we found that components of the DISC complex can be found in lipid rafts after treatment with avicin D, and that the efficient induction of apoptosis by avicin D is dependent upon intact lipid rafts and the integrity of the Fas signaling pathway. Disruption of lipid raft organization by the cholesterol-depleting compound MCD not only abolishes the clustering of Fas and its DISC complex, but reduces avicin D-induced cell apoptosis as well. Interestingly, avicin D-induced activation of the Fas pathway is not dependent on the linkage of extracellular Fas ligands or the application of agonist Fas antibodies, suggesting that avicin D may incorporate into cell membrane rafts, promoting their clustering as well as the translocation of Fas and its downstream signaling molecules into clustered rafts.

Although the exact mechanism by which avicin D induces cholera toxin capping and Fas recruitment to lipid rafts remains elusive, our data indeed indicate that avicin D rapidly induces membrane rafts, which aggregate at the cell surface. Fas and its downstream effectors are recruited into these rafts enriched in apoptotic signaling molecules, which appear to function as platforms to elicit an efficient apoptotic response. Thus, the recruitment of Fas and its downstream effectors into membrane rafts may provide a mechanism for amplifying Fas signaling by reorganizing membrane microdomains and bringing molecules together in a well-defined and reduced space, facilitating interactions among different signaling molecules and pathways [30]. Our results suggest that such rafts might be targets for therapeutic intervention. However, the results reported here also indicate that in the absence of Fas, the clustering of these rafts is not sufficient to mount an apoptotic response in avicin D-treated cells (Figure 7). Thus, rafts behave as recruitment platforms to facilitate and potentiate downstream signaling events, with Fas functioning as a critical trigger of the apoptotic process.

We previously reported that avicin parental compound F094, which includes avicin D, avicin G, and some other unknown components, can inactivate AKT [6]. As PI3K inhibition is known to induce Fas activation and is Fas-ligand independent [31], this might raise the concern that avicin D activates Fas separate of its ability to induce cholera toxin capping and recruitment of Fas and toxin to detergent resistant membrane fractions. However, we found that inactivation of AKT by avicin parental compound F094 is a later effect, 24–48 hrs after the exposure. In this study, we showed that avicin D-mediated clustering of lipid rafts and activation of Fas took place as early as 2–4 hrs after the treatment (Figure 3). In addition, as one of the components in avicin parental compound F094, avicin D has only a marginal effect on AKT inactivation [11]. Most importantly, blockage of lipid raft organization via the depletion of cholesterol by MCD substantially prevents avicin D-mediated Fas activation and cell apoptosis (Figure 4). Taken together, these data provide unambiguous evidences to prove that avicin D-induced cell apoptosis is a direct accomplishment of lipid microdomain reorganization and Fas activation.

The clinical usefulness of exogenous activation of Fas, as well as of the other major death receptors (i.e., TNF and TRAIL receptors) by their respective ligands or agonists has been hampered by toxic side effects [16], [21], [32], [33]. Systemic administration of TNF causes a severe inflammatory response syndrome that resembles septic shock [34]. Likewise, the administration of the agonistic antibody to Fas in mice causes lethal liver failure through massive hepatocyte apoptosis [35].

However, our studies showed that non-transformed normal cells are resistant to avicin D treatment [6]. This may be due to the fact that avicin D does not activate Fas extracellularly, but from an intracellular FasL-independent manner. The compound may be taken up by tumor cells but not by normal cells because of a possible difference between the membranes of normal and malignant cells. A variation in the permeability of these cells to avicin D may account for the failure to trigger the ensuing apoptotic events.

In this regard, avicin D may lead to the use of raft-dependent and intracellularly activated Fas-mediated killing in cancer chemotherapy, representing a new way to target tumor cells. Further elucidation of the mechanisms regulating avicin D uptake, intracellular Fas activation, and the formation of rafts may lead to the development of novel cancer therapeutic strategies that have few or no adverse effects on normal tissues.
